# High CFTR expression in philadelphia chromosome-positive acute leukemia protects and maintains continuous activation of BCR-ABL and related signaling pathways in combination with PP2A

**DOI:** 10.18632/oncotarget.15510

**Published:** 2017-02-19

**Authors:** Xi Yang, Tianyou Yan, Yuping Gong, Xuehua Liu, Huaqin Sun, Wenming Xu, Chunsen Wang, Duolan Naren, Yuhuan Zheng

**Affiliations:** ^1^ Department of Hematology, West China Hospital, Sichuan University, Chengdu, China; ^2^ Sichuan University-The Chinese University of Hong Kong Joint Laboratory for Reproductive Medicine, West China Second University Hospital, Chengdu, China; ^3^ Department of Hematology, Sichuan Provincial People's Hospital, Affiliated Hospital of University of Electronic Science and Technology of China, Chengdu, China

**Keywords:** CFTR, Ph^+^ acute leukemia, PP2A

## Abstract

Cystic fibrosis transmembrane conductance regulator (CFTR) is classified as an anion channel transporter of Cl^−^ and HCO3^−^. Through interactions with its PDZ domain, CFTR is capable of regulating other proteins, such as protein phosphatase 2A (PP2A). The aberrant expression and mutation of CFTR have been observed in several tumor, but not in philadelphia chromosome–positive(Ph^+^) acute leukemia, including Ph^+^ B cell acute lymphoblastic leukemia(Ph^+^ B-ALL) and chronic myelogenous leukemia blast crisis phases (CML-BC). In this study, we demonstrated the mean expression level of CFTR in Ph^+^ acute leukemia cells was markedly higher than that in Ph- B-ALL and CML-chronic phase cells. CFTRinh-172, a classic CFTR inhibitor, down-regulated the expression of CFTR, p-BCR-ABL and classical Wnt/β-catenin signaling in Ph^+^ acute leukemia cells, while imatinib had no effect on CFTR. Importantly, reduced efficacy of CFTRinh-172 was closely associated with elevated PP2A phosphatase activity. Furthermore, we confirmed an interaction between CFTR and the PP2AA subunit in K562 cells. In addition, we demonstrated CFTR and PP2AA interact in the cytosol, resulting in PP2_A_ complex inactivation and increased degradation of PP2_A_ substrates via the lysosomal/proteasome pathway. In conclusion, our results showed CFTR was highly expressed in Ph^+^ acute leukemia, which protected and maintained the continuous activation of BCR-ABL and the canonical Wnt/β-catenin signaling pathway by decreasing PP2A phosphatase activity. According to this working model of the CFTR-PP2A-BCR-ABL axis, targeting the CFTR protein will activate PP2A and may offer a new treatment strategy for Ph^+^ acute leukemia, especially for patients exhibiting high levels of CFTR expression.

## INTRODUCTION

The *BCR-ABL* oncogene causes chronic myelogenous leukemia(CML) and a fraction of pre-B cell acute lymphoblastic leukemias(pre-B-ALLs). The genetic lesion encoding the BCR-ABL fusion protein is a t(9;22) translocation termed the Philadelphia chromosome (Ph) [1]. The ABL kinase inhibitor imatinib results in stable remission for many patients with CML but is less effective in patients with Ph-positive acute lymphoblastic leukemia (Ph+ ALL)and patients in the myeloid or lymphoid blast crisis (BC) phases of CML [2], which is collectively referred to as Ph+ acute leukemia. The development of clinical resistance to tyrosine kinase inhibitors (TKIs) has prompted investigators to seek novel compounds that specifically target other signaling pathways that are essential for BCR-ABL-mediated cell survival.

The gain of oncogene function associated with the loss of tumor suppressors is widely recognized as a hallmark of cancer initiation and progression [3]. One mechanism by which a normal cell maintains the balance between tumor-inducing and tumor-suppressing signals as well as appropriate responses to extracellular stimuli is reversible protein phosphorylation [4]. In particular, protein phosphatase 2A (PP2A) has been the subject of recent investigations that have suggested its central role in cancer. A bona fide tumor suppressor protein, PP2A negatively regulates many of the signals triggered by oncogenic kinases. Likewise, impaired PP2A phosphatase activity has been linked to B-cell chronic lymphocytic leukemia(B-CLL), Ph+ B-ALL and CML-BC. Recent studies have demonstrated that PP2A phosphatase activity is markedly reduced in both CD34+ CML and CD34+/CD19+ Ph+ B-ALL bone marrow progenitors [5, 6]. PP2A inactivation results from an increased expression of SET, which was induced by BCR-ABL in a dose- and kinase-dependent manner and, like BCR-ABL, progressively increases during the transition to CML-BC. In contrast, SET down-regulation and ectopic expression of the PP2Ac subunit suppress the phosphorylation of MAPK, STAT5, Jak2 and AKT; decrease myc expression; and increase the levels of the pro-apoptotic protein BAD and the hypophosphorylated form of Rb [5, 6]. Thus, PP2A has emerged as a highly promising drug target for the development of a new series of anticancer agents, which have the potential to overcome drug resistance induced in patients by continuous exposure to kinase inhibitor monotherapy.

Cystic fibrosis transmembrane conductance regulator (CFTR) is a member of the ATP-binding cassette(ABC) transporter superfamily and is a plasmamembrane-associated cyclic AMP-activated Cl- channel that mediates the transport of Cl^−^ and HCO_3_^−^[7]. CFTR has a regulatory (R) domain that contains multiple phosphorylation sites. The transmembrane movement of Cl^−^ and HCO_3_^−^ is regulated at these sites by protein kinase A(PKA), protein kinase C (PKC), protein phosphatase 2A(PP2A), and protein phosphatase 2C(PP2C), among other proteins [8]. CFTR is widely expressed in the human respiratory, gastrointestinal, and reproductive tracts as well as in other epithelial cells and is also expressed in nerve cells, immune cells and other non-epithelial cells. The dysfunction of CFTR often manifests as abnormal external secretion in the respiratory tract, digestive tract, urogenital tract and other epithelial tissues and can lead to diseases such as cystic fibrosis (CF), secretory diarrhea, polycystic kidney disease (PKD), and infertility [9]. Recent studies have provided credible evidence that CFTR is not only an anion channel protein but also acts as a regulator that interacts with other proteins through its PDZ domain to regulate their function [10, 11]. In recent years, an enormous amount of research has shown that aberrant expression or mutation of CFTR is involved in the incidence and development of gastric cancer, colon cancer, lung cancer, breast cancer, prostate cancer, cervical cancer, ovarian cancer and other tumors [12–16].CFTR alterations differ among tumor types: in some cases, CFTR acts as a tumor suppressor gene (e.g., colon cancer and prostate cancer), whereas in others, it acts as an oncogene (e.g.,cervical cancer and ovarian cancer). Interestingly, studies of CFTR have mainly focused on solid tumors while only rarely examining hematological oncology. Related studies have been limited to investigating the expression and function of the CFTR ion channel in leukemia cells but have rarely delved into survival-related functions and mechanisms, especially regarding its interaction with other proteins [17, 18]. Kratz et al. reported a case of a child with CF who, between the ages of five weeks and five months, suffered from anemia; bone marrow examinations revealed three lines of developmental hematological defects [19]. Piro et al. [20] found that mouse bone marrow-derived hematopoietic stem/progenitor cells (HSPCs) express CFTR mRNA and protein and play important roles in hematopoiesis. These studies suggest that high CFTR expression may be involved in the development of Ph+ acute leukemia and may be a new biomarker for monitoring treatment and assessing prognosis.

To elucidate the role of CFTR in leukemia cells, we analyzed the expression of CFTR protein in normal and leukemia cells and found that the mean level of CFTR expression in Ph+ acute leukemia cells (Ph+ B-ALL and CML-blast crisis) was markedly higher than in Ph-B-ALL and CML-chronic phase cells, with HBE cells as a positive control and normal mononuclear cells (MNCs) as a normal control. Furthermore, CFTRinh-172 (a selective CFTR inhibitor) elicited significant anti-proliferative, apoptotic and cell cycle arrest phenotypes in CFTR-high cells but had little effect on normal control cells. We therefore hypothesize that CFTR may interact with PP2A—a bona fide tumor suppressor protein in Ph+ acute leukemia—to protect and maintain the continuous activation of BCR-ABL and related signaling pathways. Our data provide details of the mechanism by which high CFTR expression protects and maintains the continuous activation of BCR-ABL and the canonical Wnt/β-catenin signaling pathway by decreasing PP2A phosphatase activity in the cytosol.

## RESULTS

### CFTR was highly expressed in Ph+ acute leukemia cells

**Figure 1:**
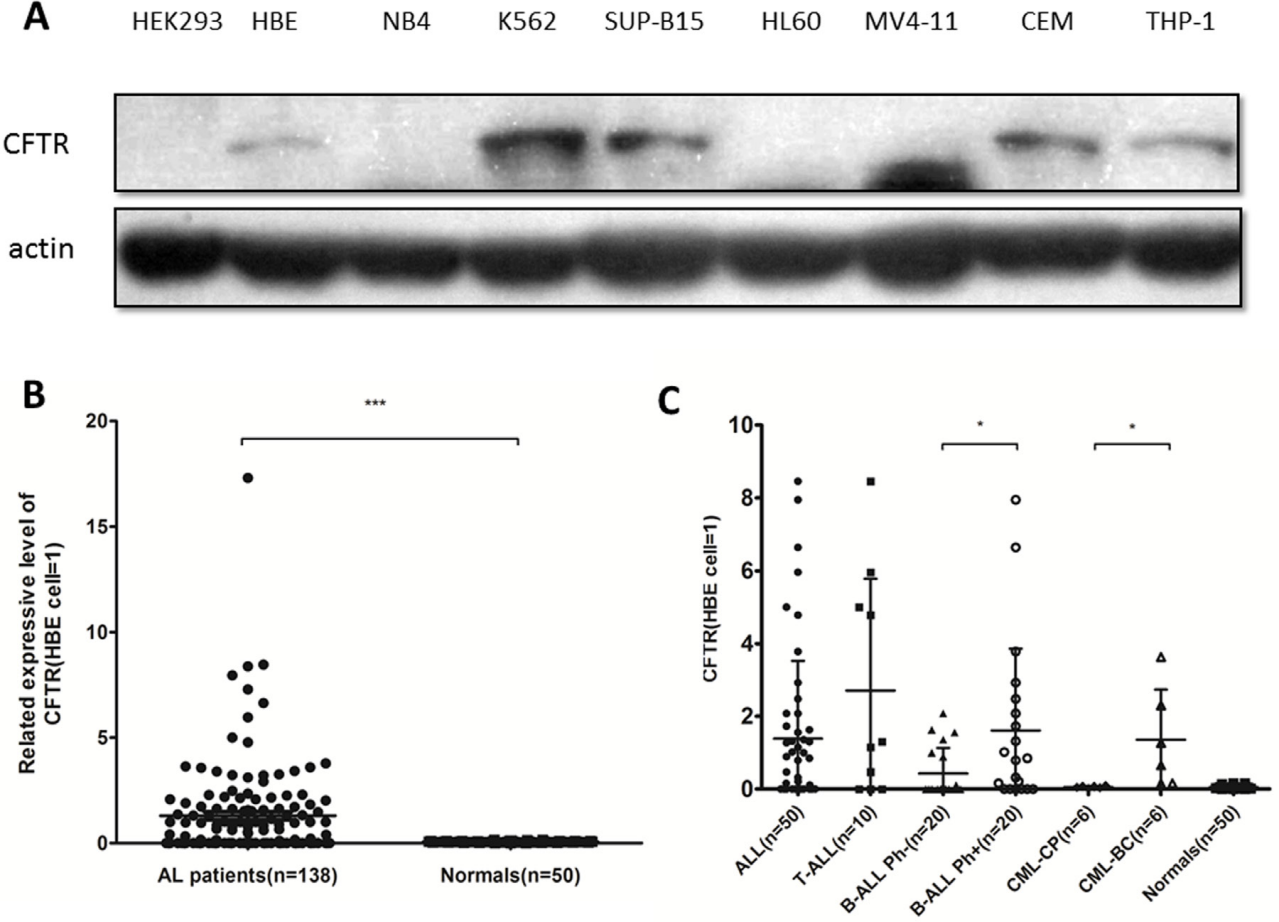
CFTR expression in leukemia cell lines and primary leukemia samples (**A**) CFTR protein expression in leukemia cell lines was detected by Western blotting with HBE as a positive control (value = 1) and HEK293 as a negative control. (**B**) CFTR protein expression in primary leukemia samples (*n* = 138) and normal human MNCs (*n* = 50) was detected by Western blotting. Significance compared with the MNC group is indicated. Data are given as the mean ± SEM relative to HBE expression. ***P < 0.001. (**C**) Among 40 cases of B-ALL and 12 cases of CML primary leukemia cells, the expression of CFTR in 20 cases of Ph+ B-ALL and 6 cases of CML-BC was higher than that in 20 cases of Ph- B-ALL and 6 cases of CML-CP. *P < 0.05.

CFTR protein is abnormally expressed in several types of tumor cells that originated as epithelial cells, but its role inhuman leukemia had not previously been described. To evaluate the role of CFTR in leukemia cells, we investigated the expression of CFTR protein in normal cells and leukemia cells, including seven common leukemia cell lines and 138 primary acute leukemia samples, by Western blotting assay. When normalized against the expression level in the normal human airway epithelial cell line HBE (a positive control, CFTR expression = 1), CFTR expression in K562 (value = 2.921) and SUP-B15 (value = 2.042) cells was higher than in other leukemia cell lines (Figure 1A).CFTR expression in normal MNCs was very low (mean value = 0.068 ± 0.005; Figure 1B); however, among the 40 cases of ALL primary leukemia cells included in the study, the expression of CFTR was higher in the 20 cases of Ph+ B-ALL (mean value = 1.613) than in the 20 cases of Ph– B-ALL (mean value = 0.432).Similarly, in 12 cases of CML primary leukemic cells, the expression of CFTR in 6 cases of CML-blast crisis (mean value = 1.360) was higher than that in 6 cases of CML-chronic phase (the mean value = 0.060). These results demonstrated that CFTR protein is highly expressed in Ph+ acute leukemia cells (Figure 1C and Table 1).

**Table 1:** CFTR expression in ALL and CML samples relative to the HBE cell line

	All ALL	T-ALL	B-ALL Ph-	B-ALL Ph+	CML-CP	CML-BC	Normal
Mean	1.386	2.712	0.432	1.613	0.060	1.360	0.068
SD	0.303	0.970	0.157	0.502	0.012	0.561	0.005
Median	0.395	1.226	0.040	0.820	0.060	0.960	0.060
Min	0.050	0.050	0.040	0.050	0.020	0.15	0.010
Max	8.460	8.460	2.080	7.950	0.100	3.630	0.160
>>normal	24	7	7	10	0	4	0
≤normal	26	3	13	10	6	2	50
Total number	50	10	20	20	6	6	50

NOTE: T-ALL: T-cell ALL samples; B-ALL: B-cell ALL samples; Ph+: all samples containing the Philadelphia chromosome; Ph−: samples not containing the Philadelphia chromosome; SD: standard deviation.

>> normal: sample values with high expression of CFTR based on the range of expression observed in normal human MNCs;

≤ normal: sample values with low expression of CFTR based on the range of expression observed in normal human MNCs.

### CFTRinh-172 exerted a significant anti-proliferative, apoptosis- inducing and cell cycle-arresting effect on CFTR-high Ph+ leukemia cells but had little effect on normal cells

**Figure 2:**
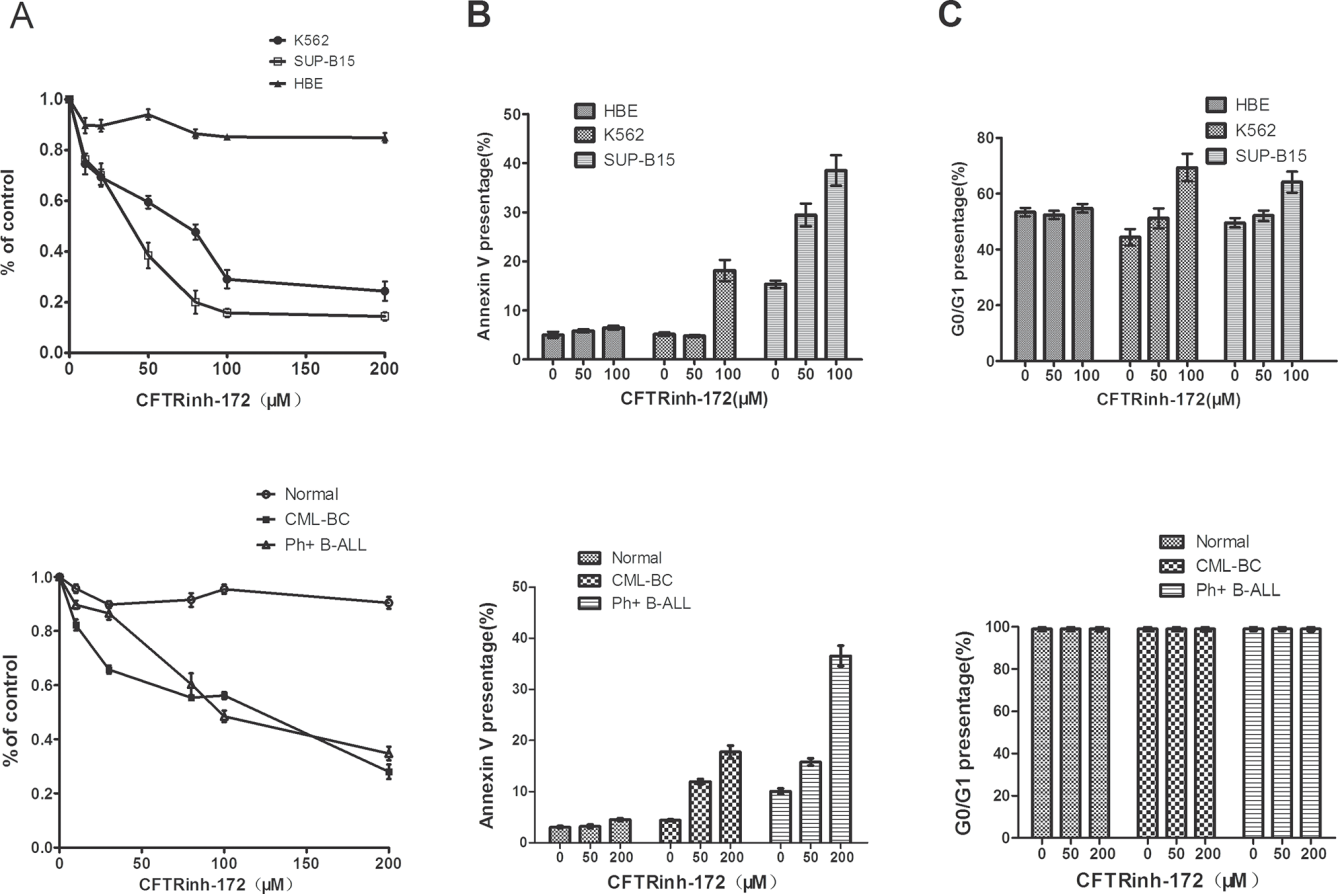
CFTRinh-172 exerted a significantly anti-proliferative, apoptotic and cell cycle-arrest effect on CFTR-high Ph+ leukemia cells but had little effect on normal cells (**A**) The inhibition of proliferation (MTT assay). (**B**) The induction of apoptosis (Annexin-V/PI FACS). (**C**) Cell cycle-arrest effect. Top: cell lines; bottom: primary leukemia cells. The data represented means ± SD of three experiments.

As described above, we found that CFTR protein is highly expressed in Ph+ ALL cells. It was therefore crucial to investigate the role of CFTR in the survival of leukemia cells. CFTRinh-172, a classic CFTR inhibitor, can stabilize the CFTR channel in its closed state by binding at or near arginine-347 on the CFTR cytoplasmic surface [21]. Based on our initial results, we used optimal drug concentrations of CFTRinh-172 (0–200 μM) to test its inhibitory effect on leukemia cells. We first assessed the proliferative activity of the K562 and SUP-B15 cell lines as well as the corresponding primary leukemia cells by MTT assay after treatment with CFTRinh-172 and further compared their diversity. We found that CFTRinh-172 (0–200 μM) exerted a significant anti-proliferative effect on the K562 and SUP-B15 cell lines (the IC_50_ values were 85.3 ± 12.5 μM and 49.1 ± 8.1 μM, respectively) but had little effect on the HBE cell line(the inhibition rate at a dose of 200μM was only 13.3%). CFTRinh-172 (0–200 μM) also had a significant anti-proliferative effect on CML-BC and Ph+ B-ALL primary leukemia cells(the IC_50_ values were 97.8 μM and 105.5 μM, respectively) but had little impact on the normal human MNCs (Figure 2A).

Cell apoptosis and cell cycle regulation are two important aspects of cell survival. We assessed the cell apoptosis and cell cycle distribution of CFTR-high leukemia cell lines and corresponding primary leukemia cells by flow cytometry after treatment with CFTRinh-172. Our results showed significant apoptosis-inducing and cell-cycle-arrest effects:1) Compared with the untreated K562 and SUP-B15 controls (5.1% and 15.6%, respectively), cells treated with CFTRinh-172 (50 μM and 100 μM) exhibited significant cell apoptosis at rates of 4.8% and 18% (50 μM) and 29.4% and 39.5% (100 μM), respectively, corresponding to changes of −0.3% and 12.9% (50 μM) and13.8% and 23.9% (100 μM),respectively (Figure 2B). Little effect was observed on the HBE cell line. 2) Compared with untreated K562 and SUP-B15 controls(G_0_/G_1_ phase ratios were 47.5% and 47.7%, respectively), CFTRinh-172 treatment (50 μM and 100 μM) led to G_0_/G_1_ phase stagnation, with G0/G1 phase ratios of 50.6% and 66.1% (50 μM)and 50.3% and 60.5% (100 μM), respectively, corresponding to increases of 3.1% and 18.6% (50 μM) and 2.6% and 12.8% (100 μM), respectively (Figure 2C). Little effect was observed on the HBE cell line.3) The effect of CFTRinh-172 (0–200 μM) on apoptosis in CML-BC and Ph+ B-ALL primary leukemia cells was similar to that observed for the K562 and SUP-B15 cell lines, but little effect was observed on normal human MNCs (Figure 2B). 4) Cell cycle analysis revealed no difference in primary leukemia cells because these primary cells were unable to proliferate *in vitro* (Figure 2C).

### Similar to imatinib, CFTR inhibition down-regulated the expression and activity of BCR-ABL and the canonical Wnt/β-catenin pathway in CFTR-high Ph+ acute leukemia cells

**Figure 3:**
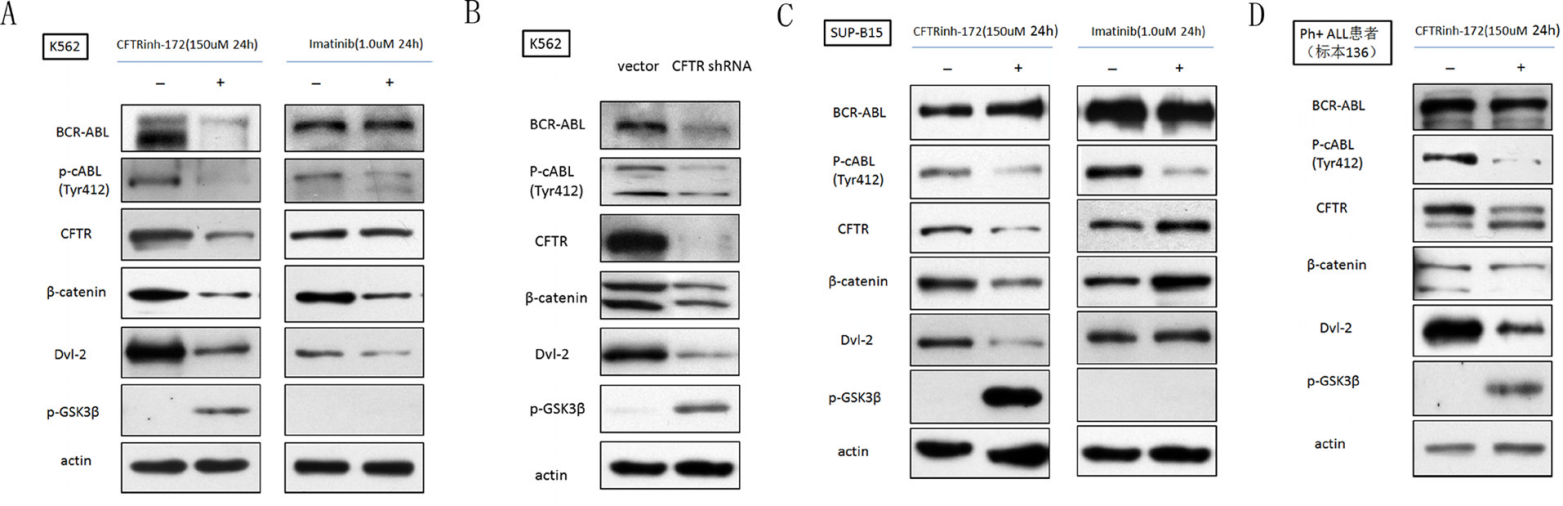
CFTR inhibition down-regulated the expression and activity of BCR-ABL and canonical Wnt/β-catenin signaling in CFTR-high Ph+ acute leukemia cells (**A**) CFTRinh-172 and imatinib down-regulated CFTR, BCR-ABL and classic Wnt/β-catenin signaling in K562 cells. (**B**) CFTR shRNA induced a similar effect on CFTR, BCR-ABL and classic Wnt/β-catenin signaling in K562 cells compared with CFTRinh-172. (**C**) CFTRinh-172 and imatinib down-regulated CFTR, BCR-ABL and classic Wnt/β-catenin signaling in SUP-B15 cells. (**D**) CFTRinh-172 and imatinib down-regulated CFTR, BCR-ABL and classic Wnt/β-catenin signaling in a Ph+ B-ALL primary sample.

The persistent activation of BCR-ABL is essential for the survival of Ph+ leukemia cells, and canonical Wnt/β-catenin signaling is one of the key pathways involved in maintaining the growth of leukemia cells. Because the CFTR inhibitor showed a strong anti-leukemia effect, we sought to determine the effect of down-regulating CFTR expression on BCR-ABL and classic Wnt/β-catenin signaling. K562 cells, SUP-B15 cells and a Ph+ B-ALL primary sample were treated with CFTRinh-172(150 μM, 24 h), and a Western-blot assay was performed with t-BCR-ABL, p-BCR-ABL, Dvl-2, p-GSK3β, β-catenin and β-actin antibodies. As expected, when CFTR was reduced by CFTRinh-172, canonical Wnt/β-catenin signaling was significantly down- regulated(Dvl-2 and β-catenin were significantly down-regulated, and p-GSK3β was significantly up-regulated; Figure 3A, 3C, 3D). Consistent with the results obtained using imatinib, CFTRinh-172 led to a significant reduction in p-BCR-ABL in both K562 and SUP-B15 cells as well as significant down-regulation oft-BCR-ABL in K562 cells (Figure 3A) but not in SUP-B15 cells (Figure 3C).Notably, imatinib had no effect on CFTR expression but did result in the down-regulation of p-BCR-ABL and classic Wnt/β-catenin signaling, implying that CFTR protein is located upstream of BCR-ABL in the signaling regulatory networks active in Ph^+^ acute leukemia cells. To corroborate the results obtained for the CFTR inhibitor, CFTR was knocked down by shRNA interference in the K562 cell line; a similar result is shown in Figure 3B.

### CFTR inhibition down-regulates p-BCR-ABL and canonical Wnt/β-catenin signaling in K562 cells

**Figure 4:**
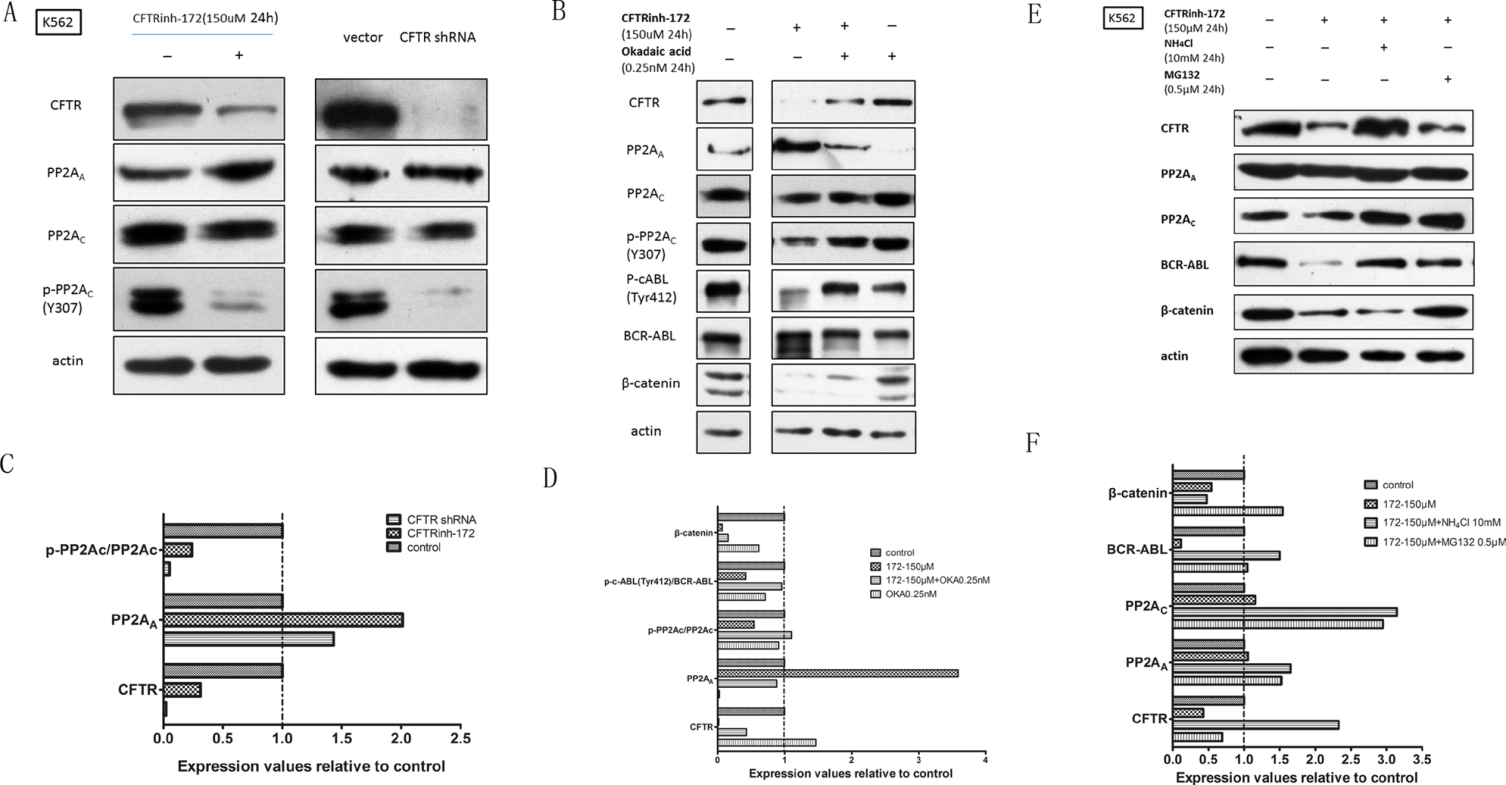
CFTR inhibition down-regulated p-BCR-ABL and canonical Wnt/β-catenin signaling by restoring PP2A activity in the K562 cell line (**A**) CFTRinh-172 (150 μM, 24 h) and CFTR shRNA interference increased the level of the PP2A_A_ subunit and significantly reduced p-PP2A_C_ (Y307)/PP2A_C_. (**B**) Okadaic acid (OKA, a PP2A inhibitor) was used to verify the role of PP2A in CFTRinh-172-mediated regulation. The effect of OKA combined with CFTRinh-172 on CFTR, PP2A, BCR/ABL and β-catenin was examined by Western blotting. (**C**) Relative expression values for the data shown in Figure 5A were calculated by semi-quantitative analysis using Quantity One Soft software, which compared differences among the different treatment groups. Each stripe was calculated three times, and average values were used for data analysis. (**D**) Relative expression values for the data shown in Figure 5B were calculated according to the method described in C. (**E**) To confirm a change in PP2A activity, MG132(a proteasome inhibitor, 0.5 μM, 24 h) and NH4Cl (a lysosome inhibitor, 10 mM, 24 h) were used to analyze CFTR-mediated degradation pathways for CFTR, PP2A, BCR-ABL and β-catenin. (**F**) Relative expression values for the data shown in Figure 5E were calculated according to the method described in C.

What are the mechanisms mediating the phosphorylation of p-BCR-ABL and related signaling molecules following CFTR knockdown? An emerging body of evidence suggests a central role for PP2A in Ph+ acute leukemia, whereas BCR-ABL and related signaling molecules are both substrates of PP2A. We therefore speculated that PP2A is involved in the CFTRinh-172-mediated inhibitory effect on p-BCR-ABL and classic Wnt/β-catenin signaling. To test our hypothesis, PP2A subunits were examined by Western-blot assay following CFTR knockdown in the K562 cell line. Our results confirmed that CFTRinh-172 (150 μM, 24 h) and CFTR shRNA interference increased the level of the PP2A_A_ subunit and significantly reduced the p-PP2A_C_(Y307)/PP2A_C_ rate(the phosphorylation of Y307 of the PP2A_C_ subunit was responsible for PP2A inactivation), indicating that CFTR knockdown could enhance PP2A activity (Figure 4A). In addition, we used okadaic acid(OKA, a PP2A inhibitor) to verify this hypothesis. Our results showed that CFTRinh-172 (150 μM) in combination with OKA (0.25 nM) inhibited the CFTRinh-172-mediated up-regulation of the PP2A_A_ subunit, significantly increased the p-PP2A_C_(Y307) /PP2A_C_ rate, and partially restored the levels of CFTR, p-BCR-ABL and β-catenin following CFTRinh-172-mediated down-regulation, providing strong evidence for the inhibition of PP2A by CFTR (Figure 4B). In addition, we further analyzed CFTR-mediated degradation pathways by using MG132 as a proteasome inhibitor and NH_4_Cl as a lysosome inhibitor for BCR-ABL and β-catenin expression to confirm the change in PP2A activity. We found that β-catenin was degraded by the ubiquitin-dependent pathway, whereas BCR-ABL degradation depended on the ubiquitin-dependent and lysosome-dependent pathway (Figure 4E). Relative expression values for the results shown in Figure 4A, 4B and 4E were calculated by semi-quantitative analysis using Quantity One Soft software, which was used to compare differences among the different treatment groups (Figure 4C, 4D, and 4F). Together, these results indicate that CFTR inhibits PP2A activity, thereby protecting and maintaining the continuous activation of BCR-ABL and canonical Wnt/β-catenin signaling.

### The cytosolic interaction between CFTR and the PP2AA subunit is blocked by CFTRinh-172

**Figure 5:**
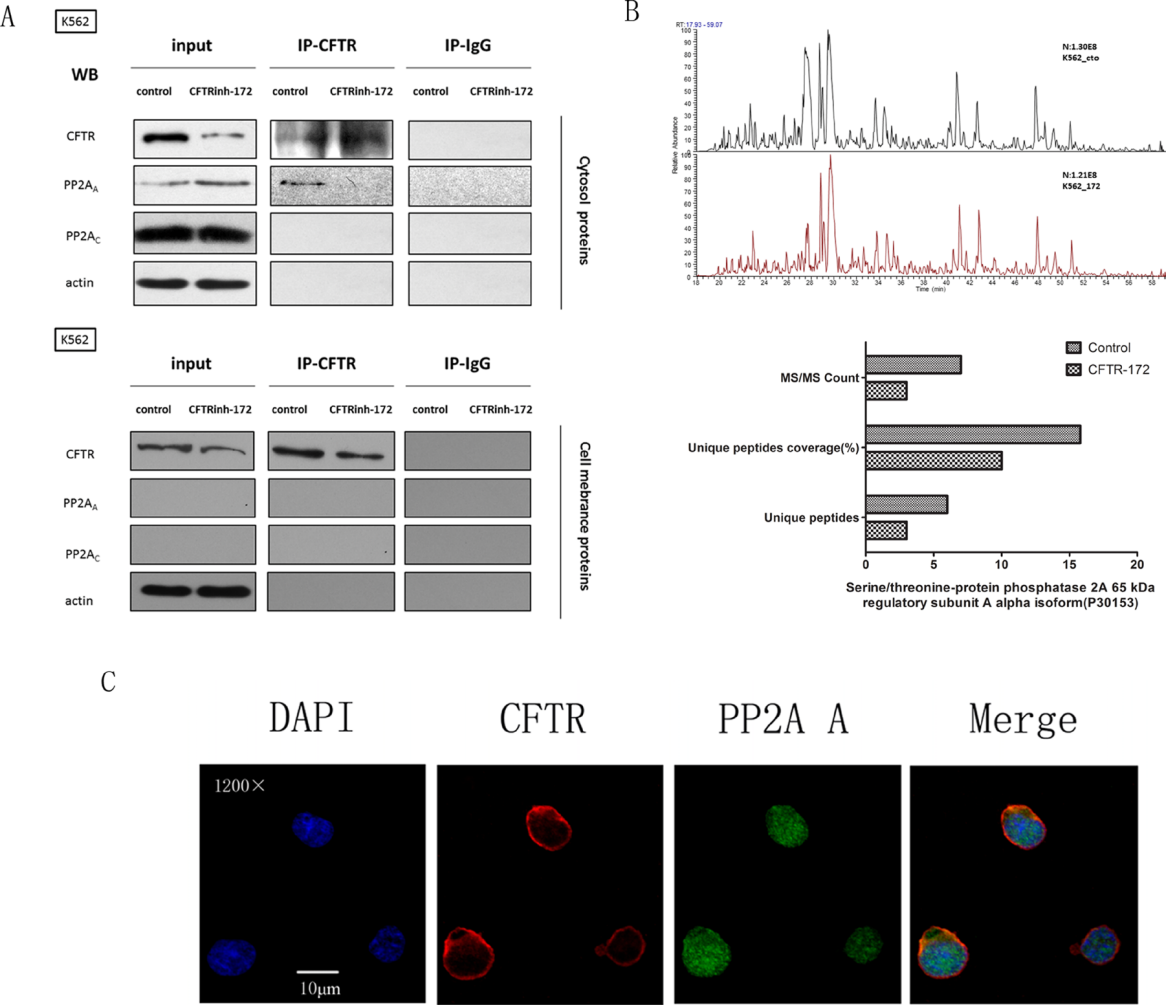
The interaction between CFTR and PP2A_A_ subunits was interrupted by CFTRinh-172 (**A**) K562 cells were exposed to CFTRinh-172 (150 μM, 24 h) and were lysed to extract the cytosolic and membrane-associated proteins. PP2A subunits were immunoprecipitated with CFTR and detected by Western-blot assay using monoclonal antibodies specific to the CFTR, PP2A_A_ subunit and PP2A_C_ subunits. (**B**) LC-MS analysis confirmed the presence of the PP2A_A_ subunit in IP-CFTR products between 50 kd and 70 kd and revealed a decrease in PP2A_A_ subunit in IP-CFTR products after treatment with CFTRinh-172. The column graph shows the signal attributed to the PP2A_A_ subunit(PR65) in two report lists. Top: the control group; bottom: the CFTRinh-172 treatment group. (**C**) CFTR and PP2A_A_ subunit exhibited marked co-localization in the cytosol as detected by IF co-localization analysis. K562 cells were stained with mAbs against CFTR (red) and the PP2A_A_ subunit (green) as well as DAPI(blue). Fluorescence images were collected using a confocal microscope. Orange indicates the colocalization of CFTR and PP2A_A_(magnification, ×1200).

Previous studies have demonstrated a direct and functional interaction between CFTR and PP2A: it is possible that the regulatory domain (R domain) of CFTR is dephosphorylated by PP2A *in vitro*. Overexpression of the interacting domain of the PP2A_A_ subunit(PR65) in the Caco-2 cell line (heterogeneous human epithelial colorectal adenocarcinoma) as well as treatment with OKA resulted in the prolonged deactivation of the chloride channel [22]. However, this previous study focused on how PP2A affected the function of the CFTR anion channel while ignoring how CFTR impacted PP2A activity. Interestingly, our research showed that CFTR inhibited PP2A anti-tumor activity, revealing its central role in Ph+ acute leukemia. We therefore proposed a hypothesis: CFTR and PP2A interacted through a special domain or subunit in Ph+ acute leukemia cells. To test this hypothesis, we extracted the cytosol and cell membrane proteins of K562 cells and performed co-immunoprecipitation (Co-IP) analyses with a CFTR antibody. As shown in Figure 5A, we found that CFTR can combine with the PP2A_A_ subunit (rather than the PP2A_C_ subunit), and PP2A_A_ subunit binding with CFTR was reduced in the cytosol following treatment with CFTRinh-172. However, we did not detect the expression of the PP2A_A_ and PP2A_C_ subunits in the cell membrane fraction in either the input group or the IP-CFTR group. These results suggest that the interaction between CFTR and PP2A exists and occurs in the cytosol but not at the cell membrane. Furthermore, we confirmed this interaction in the K562 cell line: using LC-MS analysis, we affirmed the presence of the PP2A_A_ subunit in IP-CFTR products between 50 kd and 70 kd and demonstrated a decrease in PP2A_A_ subunit level among IP-CFTR products after treatment with CFTRinh-172 (Figure 5B).These findings are consistent with the results of our Co-IP analysis: CFTR and the PP2A_A_ subunit showed obvious co-localization in the cytosol based on IF co-localization analysis (Figure 5C). Taken together, these results and previous studies support an interaction between CFTR and PP2A in the cytosol of K562 cells in contrast with the membrane interaction in epithelial cells.

## DISCUSSION

Despite recent interest in the correlation between abnormal CFTR protein (including expression and mutations) and various cancers, studies of the role of CFTR in cancer pathogenesis, particularly leukemia, remain limited. Our study presents three principal findings. First, CFTR expression is significantly higher in Ph+ acute leukemia cell lines and patient samples than in Ph– B-ALL and CML-chronic phase cells. Second, high CFTR expression is crucial for the survival of Ph+ acute leukemia cells. Finally, high CFTR expression inhibits the phosphatase activity of PP2A to protect and maintain the continuous activation of BCR-ABL and canonical Wnt/β-catenin signaling via the interaction with the PP2A_A_ subunit in the cytosol of Ph+ acute leukemia cells. These findings are ground breaking and have great clinical relevance for the treatment and prognosis of Ph+ acute leukemia.

Past CFTR-related studies have focused on epithelial cells, and few studies have involved hematopoietic cells. Mature CFTR protein function is closely associated with post-translational modification. Therefore, our study did not detect the change in the gene expression level of CFTR, but did detect a change in protein level with a Western-blot assay. Our results demonstrated that CFTR expression was significantly higher in Ph+ acute leukemia cells than that in normal controls, Ph– B-ALL cells and CML-CP cells. Importantly, not all of the leukemia cells expressed elevated CFTR protein; CFTR expression in a small fraction of leukemia cells was similar to that observed for normal cells. These results suggest that CFTR expression may be random in different leukemia patients, although high expression of CFTR is directly or indirectly linked with BCR-ABL. We therefore selected Ph+ acute leukemia as a research model.

**Figure 6:**
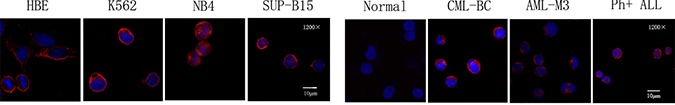
CFTR protein was primarily localized in the cytoplasm of leukemia cells and normal MNCs K562, NB4, SUB-B15, HBE, normal MNCs, and primary samples with CML-BC, AML-M3 and Ph+ B-ALL were stained with mAb against CFTR (red) and DAPI (blue). Fluorescence images were collected using a confocal microscope. (magnification, ×1200).

CFTR is an ion channel, and mature CFTR protein is mainly localized to cell membranes in various epithelial cells. Unexpectedly, we found that CFTR protein was located in the cytosol in leukemia cells and normal MNCs (Figure 6). We also found that CFTR still functioned as an ion channel in these cells based on the cytosolic pH values detected (it has been reported that CFTR regulates cytosolic pH values via mediating HCO_3_^−^transport): in leukemia cells, cytosolic pH values were reduced when exposed to CFTRinh-172 at low concentrations (20–50 μM), but there was no noticeable impact on HBE cells and normal human MNCs. Higher CFTR expression resulted in greater sensitivity to cytosolic pH in response to CFTRinh-172(data not shown). We further demonstrated that high concentrations of CFTRinh-172 (100–200 μM) resulted in significant anti-proliferative, apoptotic and cell cycle-arrest effects in CFTR-high leukemia cells but had little effect on normal cells. These results suggest that CFTR plays an essential role in the survival of CFTR-high leukemia cells.

In recent years, it has become apparent that Wnt/β-catenin signaling powerfully regulates normal hematopoiesis, and its deregulation is involved in the development of leukemia [23], although the underlying mechanisms remain unclear. Wnt/β-catenin signaling is divided into classical and non-classical types. For simplicity, this research focused on the classical Wnt/β-catenin signaling pathway, which involves Dvl, GSK3β, β-catenin and members of the T-cell factor(Tcf)/lymphocyte-enhancer binding factor(Lef) family. Numerous studies have confirmed that BCR-ABL induce the aberrant activation of classical Wnt/β-catenin signaling [24]. In our study, we found that p-BCR-ABL and classical Wnt/β-catenin signaling were significantly down-regulated (i.e., Dvl-2 and β-catenin were significantly down-regulated and p-GSK3β was significantly up-regulated) when CFTR protein was reduced by CFTRinh-172 and shRNA in Ph+ acute leukemia cells. This finding provides important evidence that CFTR protects and maintains the continuous activation of BCR-ABL and canonical Wnt/β-catenin signaling.

PP2A has been the subject of recent investigations that reveal its central role in cancer. By negatively regulating many of the signals triggered by oncogenic kinases, PP2A is a bona fide tumor suppressor protein [25].Impaired PP2A phosphatase activity has been linked to the development of B-cell CLL, Ph+ B-ALL and CML-BC cells. Remarkably, drugs such as forskolin, 1,9-dideoxy-forskolinand FTY720, which lead to PP2A activation, effectively antagonize leukemogenesis in both *in vitro* and *in vivo* models of CML and Ph+ B-ALL(see the introduction). Furthermore, the interaction between CFTR and PP2A has been confirmed in previous studies: Lin et al. [26] found that PP2A is a relevant CFTR phosphatase in epithelial tissues and regulates CFTR channel function via direct interaction involving the COOH terminus of CFTR and the PP2A B’ regulatory subunit. In addition, Vastiau A. et al. [22] demonstrated a direct and functional interaction between CFTR and PP2A in Caco-2 cells and found that an interaction occurred between the R domain of CFTR and the PP2A_A_ structure subunit. Similarly, our results support that CFTR protects and maintains the activity of BCR-ABL and classical Wnt/β-catenin signaling by interacting with the PP2A_A_ subunit in CFTR-high Ph+ acute leukemia cells, representing the major mechanism by which CFTR protein interacts with signaling regulatory networks. On this point, we observed a similar mechanism of interaction to that reported by Vastiau A., however, other modes of interaction have also been reported. Our study is the first to show how CFTR protein regulates the activity of PP2A in Ph+ acute leukemia cells, whereas the other two studies did not report this effect: high CFTR expression inhibited the phosphatase activity of PP2A to protect and maintain the continuous activation of BCR-ABL and classic Wnt/β-catenin signaling via the interaction with PP2A_A_ subunit in the cytosol of Ph+ acute leukemia cells; The persistent activation of BCR-ABL further inhibited the anti-leukemia activity of PP2A, creating a feedback loop promoting CML-BC transformation and the progression of Ph+ B-ALL. According to these results, we may consider CFTRinh-172 to be a PP2A activator that exerts an anti-leukemia effect. Other CFTR-related regulatory mechanisms, such as CFTR and BCR-ABL, CFTR and SET, remain to be elucidated.

In conclusion, based on this working model of the CFTR-PP2A-BCR-ABL axis, CFTR protein is a PP2A activator that represents a new treatment strategy for Ph+ acute leukemia, especially for patients exhibiting high CFTR expression. In addition, CFTR will be the focus of future research into other types of leukemia and lymphoma.

## MATERIALS AND METHODS

### Reagents

CFTRinh-172(a selective CFTR inhibitor) from Sigma(USA), Imatinib provided by Novartis(China), MG132(a proteasome inhibitor) from Sigma (USA), NH_4_Cl(a lysosome inhibitor) provided by Sigma (USA)and okadaic acid(OKA, a PP2A inhibitor) from Beyotime(China) were prepared in DMSO, stored at −20°C, and diluted to suitable concentrations with RPMI 1640 before use. MTT was purchased from sigma(USA), diluted to 5 mg/mL, and stored at −20°C.

### Cell lines and cell culture

There was seven kinds of leukemia cell lines in this study: SUP-B15(a human Ph+ ALL cell line expressing P190 fusion protein) was purchased from the American Type Culture Collection (ATCC, USA, CRL-1929) and was maintained in IMDM medium(Gibco, Paisley, UK) supplemented with 10% fetal bovine serum (FBS) and penicillin/streptomycin in 5% CO_2_ and humidified atmosphere at 37°C. K562( a human CML-BC cell line expressing P210 fusion protein), CEM(a human acute lymphoblast leukemia cell line), NB4(an human acute promyelocytic leukemia cell line expressing PML/RARα fusion protein), HL-60(an human acute promyelocytic leukemia cell line), THP-1(a human acute monocytic leukemia cell line), and MV4-11(a biphenotypic B myelomonocytic leukemia) was supplied from Hematology Laboratory (Department of Hematology, West China Hospital, Sichuan University, China, 610041) and were maintained in RPMI 1640 medium(Gibco, Paisley, UK) supplemented with 10% fetal bovine serum (FBS) and penicillin/streptomycin when required in 5% CO_2_ and humidified atmosphere at 37°C. HEK293(a human embryonic kidney epithelial cell line) and HBE(a human respiratory epithelial cell line) were also supplied from Hematology Laboratory and cultured in DMEM medium(Gibco, Paisley, UK) supplemented with 10% fetal bovine serum (FBS) and penicillin/streptomycin when required in 5% CO_2_ and humidified atmosphere at 37°C.

### Primary human leukemia samples

With written informed consent and approval from local ethics committees, peripheral blood or marrow samples were collected from 138 patients with acute leukemia who had been diagnosed at the Department of Hematology, West China Sichuan University. Most patients were being treated following an initial diagnosis, whereas others were suffering from a relapse. The cohort included 20 patients with Ph+ B-ALL, 20 patients with Ph- B-ALL, 6 patients with CML-CP, and 6 patients with CML-BC. In addition, 50 normal human samples were collected for use as the normal control. Peripheral blood or marrow mononuclear cells (MNCs) were isolated using ficoll density gradient centrifugation (TBD Science, Tianjin, China), and isolated MNCs were cultured as described for the K562 cell line.

### Western blotting and antibodies

Whole-cell extracts were prepared in RIPA lysis buffer (20 mM Tris, pH 7.4, 250 mM NaCl, 2 mM EDTA, pH 8.0, 0.1% Triton-X100, 0.01 mg/mL aprotinin, 0.005 mg/mL leupeptin, 0.4 mM PMSF, 4 mM NaVO_4_). The cytosol and cell membrane protein extraction was performed according to the kit of Membrane and Cytosol Protein Extraction Kit(Beyotime Biotechnology, China,#P0033). Quantitated proteins were loaded on 8–12% sodium dodecyl sulfate–polyacrylamide gel. After electrophoresis, the total proteins were electrotransferred to PVDF membrane. The following antibodies were used: the rabbit monoclonal antibodies to BCR-ABL, p-cABL(Y412), Dvl-2, β-catenin(Cell Signaling Technology, USA); the mouse monoclonal antibody to CFTR(Abcam, USA, #ab2784) and the rabbit polyclonal antibody to CFTR(Abcam, USA, #ab2916); the mouse monoclonal antibody to β-actin from Zen BioScience(Chengdu, China). The membranes were incubated with the above primary antibody overnight at 4°C.Consequently, it was washed and incubated with horse radish peroxidase (HRP)-conjugated secondary antibody for one hour at room temperature and finally detected by enhanced chemiluminescence (ECL) detection system and film (Bio-Rad Laboratories Inc., Hercules, CA, USA) according to the manufacturer's instruction. β-actin was used as an endogenous control to standardize the amount of the sample proteins.

### Cell viability assays by MTT

Cell viability was analyzed asdescribed in our previous studies [27].

### Cell cycle analysis

A cell-cycle assay kit(Beckman Coulter, USA) was use to prepare the cell samples and was performed according to the published protocol. Following incubation at 37°C for 15 minutes, the samples were analyzed in a Galliosflow cytometer (Beckman Coulter, USA) equipped with the appropriate software.

### Apoptosis assays

Cytofluorometric analysis of the apoptotic cell fraction was performed by measuring the uptake of Annexin V (KeyGEN biotech, Nanjing, China) and PI (Sigma-Aldrich Corporation, USA) according to the published protocol [27].

### CFTR shRNA knockdown

We used the HuSH-29 shRNA system(Origene, USA) for knockdown experiments. pGFP-V-RS empty vector and a CFTR shRNA(Origene, USA) vector were obtained from the Joint Laboratory of Reproductive Medicine, Sichuan University-Chinese University of Hong Kong. For knockdown experiments, 1 mg DNA was transfected into cells using Lipofectamine 2000(Invitrogen, USA) according to the manufacturer's protocol and selected by 1 mg/ml puromycin for 2–3 weeks. The stable transfected cells were cultured in medium containing 0.5 mg/ml puromycin. The efficacy of the knockdown treatment was assessed by Western blot assay for CFTR expression.

### Co-immunoprecipitation(Co-IP) and liquid chromatography-mass spectrometry(LC-MS)

K562 cells were re-suspended in NP-40lysis buffer(20 mM TrisHCl, PH 8.0, 137 mM NaCl, 10% glycerol, 1% NP-40, 2 mM EDTA, 1:100 Protease Inhibitor Cocktail; see www.abcam.com/technical), vortexed repeatedly, and physically disrupted on ice for more than 15 min. The lysates were centrifuged at 12,000 rpm for 20 min to remove unbroken cells and lytic cell organelles. The supernatants were collected and their protein contents quantified using a BCA kit (Pierce). CFTR was immunoprecipitated using a rabbit monoclonal antibody against CFTR (Abcam, USA, #ab2916) or isotype-matched control antibodies covalently conjugated to PureProteome Protein G Mag Beads (Millipore, USA, #LSKMAGG02) according to published methods. Finally, bound proteins were denatured at 95°C for 10min and then analyzed by Western blotting using specific antibodies for PP2A subunits to investigate the interaction between CFTR and PP2A. In addition, the denatured bound proteins were separated by SDS-PAGE electrophoresis, cut out at the site corresponding to 50–60 kd in the gel, and stored at −30°C. The gel samples were sent to our collaborators for LC-MS analysis to identify the bound proteins.

### Immunofluorescence(IF) co-localization analysis

Slides mounted with several cell lines and primary cell types were fixed in 4% paraformaldehyde for more than 10 min, blocked for 30 min with PBS/0.3%TritonX-100/1%BSA, and incubated at 4°C overnight in mouse monoclonal antibody against CFTR and rabbit monoclonal antibody specific to the PP2A_A_ subunit or non-immune mouse IgG at a 1/100 dilution. For immunodetection, the slides were incubated with Alexa Fluor^®^ 594(red)- and/or AlexaFluor^®^488(green)-conjugated secondary antibody at a dilution of 1:500 and then stained with DAPI(blue)at a 1/200 dilution to label nuclei. Finally, images were obtained using a confocal microscope (Olympus FV1000) and analyzed using FV10-ASW 4.0 Viewer Soft software.

### Determination of cytosol pHi values

The cytosol pH_i_ values of cells were assessed using a full-wave length fluorescence spectrometer (CLARIOstar, Germany) equipped with the pH-sensitive fluorescent probe BCECF-AM (Beyotime Biotechnology, China, S1006). Briefly, cells (1 × 10^6^−10^7^) were incubated with the pH-sensitive fluorescent probe BCECF-AM (2.5 μM) for 30 min at 37°C in HEPES buffer (5 mM KCl, 153 mM NaCl, 5 mM glucose, and 20 mM HEPES, pH7.4). After being washed once with HEPES buffer, the cells were re-suspended in HEPES and were treated with or without various concentrations of CFTRinh-172 for 24 or 48 hours. The labeled cells were analyzed at excitation wave lengths of 440 nm and 490 nm, and the ratio of the fluorescence at 535 nm was recorded. To calibrate the fluorescence, BCECF-AM-loaded cells were suspended in pH 6.4, 6.6, 6.8, 7.0, 7.2, and 7.4 calibration buffers (130 mM KCl, 10 mM NaCl, 1 mM MgSO_4_, and 10 mM Na-MOPS), and 1.0 μM Triton-X100 was added to equilibrate the external and internal pH values. The relative fluorescence ratio values were plotted against corresponding pH_i_ values, which enabled the determination of the unknown pH_i_. With an increase in pH_i_, the optical density of the BCECF-AM solution increased linearly. From this curve, it was possible to estimate the pH_i_ of cells loaded with BCECF-AM.

### Data analysis and statistics

Statistical analysis of the data was performed using GraphPad Prism version 5.0 for Macintosh (GraphPad Software, San Diego, CA). Data are presented as the mean±SEM. Student's unpaired *t-test* (two tailed) was used for the statistical analysis of two groups. Differences between groups were analyzed using SPSS (New York, NY, USA). A *P-value* < 0.05 was considered statistically significant.

### Study approval

All primary samples were collected after obtaining ethics committee approval and informed consent from all patients.
